# Treatment of a Complex Pressure Ulcer Using a Synthetic Hybrid-Scale Fiber Matrix

**DOI:** 10.7759/cureus.14515

**Published:** 2021-04-16

**Authors:** Karena Herron

**Affiliations:** 1 Wound and Hyperbaric Oxygen, MultiCare Auburn Internal Medicine, Auburn, USA

**Keywords:** infection, exposed bone, sacral pressure injury, electrospun matrix, pressure ulcer

## Abstract

Pressure ulcers are caused by sustained pressure, friction, or shear on the skin which limits blood flow to the dermis and surrounding tissue. Symptoms include redness and ulceration and may result in a chronic wound. This ailment affects three million adults in the United States, and is a major burden to the healthcare system. Pressure ulcers are associated with pain and immobility that substantially lower the patient’s quality of life, resulting in longer and more frequent hospitalization and a higher risk of mortality. As the severity of the pressure ulcer increases, it becomes more challenging for clinicians to successfully treat the wound and achieve healing objectives. Treatment of a pressure ulcer is more challenging when the wound is associated with infection, heavy exudate, inflammation, high enzymatic activity, and exposed bone and/or muscle. An advanced technology that may provide a new solution for the treatment of pressure ulcers is the use of a biodegradable synthetic hybrid-scale fiber matrix. The synthetic hybrid-scale fiber matrix provides a porous scaffold for cellular infiltration and vascularization that leads to granulation tissue formation and re-epithelialization, before completely resorbing via hydrolysis. In this case report, a patient who had a large sacral pressure ulcer present for over 16 years with two exposed spinal segments and exposed peritoneum was treated with the hybrid-scale fiber matrix. A total of nine applications of the material were applied over 11 weeks. The wound demonstrated significant healing, wound exudate was notably reduced, and no complications were observed. New epithelial tissue formation, tissue coverage over the exposed bone, infection management, and wound exudate reduction were observed to the extent that the patient was discharged from the hospital. Overall, the excellent healing response achieved on this 16-year-old chronic wound suggests that the synthetic hybrid-scale fiber matrix may be a good treatment option for difficult-to-heal pressure ulcers.

## Introduction

Pressure ulcers affect three million adults in the United States and are a major burden to the healthcare system [1]. The treatment cost of a pressure ulcer varies from $37,800 to $70,000 [1], and the total annual cost of pressure ulcer treatment in the United States is approximately $25 billion [2]. Pressure ulcers, which form due to sustained pressure, friction, or shear on the skin resulting in ulceration and/or development of a chronic wound, are associated with pain and immobility that substantially lower the patient’s quality of life, resulting in longer and more frequent hospitalization, as well as a higher risk of mortality. The risk of pressure ulcer formation is significantly higher in elderly patients or when comorbidities such as diabetes mellitus and vascular disease exist [3]. As the severity of the pressure ulcer increases, it becomes more challenging for clinicians to successfully treat the wound and achieve healing objectives. Treatment of a pressure ulcer is more challenging when the wound is associated with infection, heavy exudate, inflammation, high enzymatic activity, and exposed bone and/or muscle.

An advanced technology that may provide a new solution for the treatment of pressure ulcers is the use of a fully resorbable synthetic hybrid-scale fiber matrix. It is composed of synthetic materials that are resistant to enzymatic/bacterial degradation and persist in the wound bed to support the healing process prior to completely resorbing via hydrolysis [4]. The electrospun hybrid-scale fiber matrix is composed of two synthetic biocompatible polymers, polyglactin 910 and polydioxanone. The synthetic hybrid-scale fiber matrix provides a porous scaffold for cellular infiltration and vascularization that leads to granulation tissue formation and re-epithelialization [4].

The goal of this single case study was to evaluate the clinical efficacy of the synthetic hybrid-scale fiber matrix to treat a patient with a challenging, refractory 16-year-old pressure ulcer.

## Case presentation

The patient was a 63-year-old Hispanic male who was living in a group home. He had multiple comorbidities including type 2 diabetes mellitus, hyperthyroid, rectal fistula, with a previous colostomy, hemipelvectomy, and urostomy surgeries. He was paralyzed due to a gunshot injury to the C10-C11 intervertebral discs. He had an abdominal abscess which resulted in a rectal fistula that was draining into the wound. A catheter was implanted to divert the drainage.

**Figure 1:**
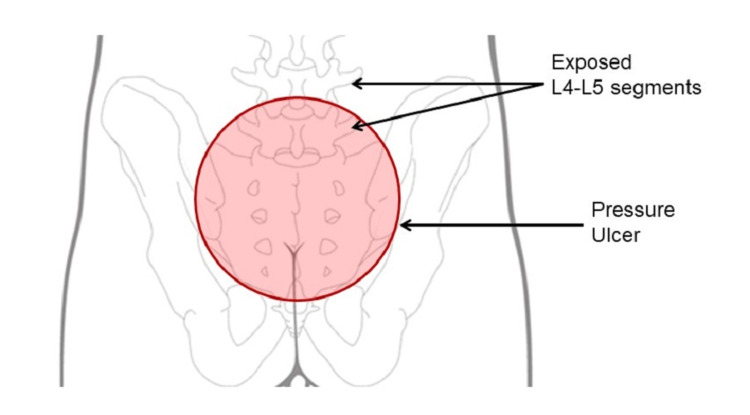
Schematic drawing of the sacral pressure ulcer.

**Figure 2:**
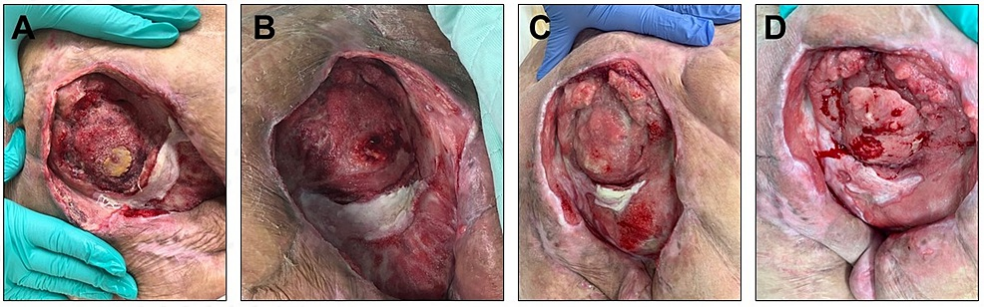
Sacral pressure ulcer. (A) The original wound size was 15 × 11 × 5 cm. (B) After application of the synthetic hybrid-scale fiber matrix, bone and spinal column were covered with new tissue. (C, D) Wound size and exudate were significantly reduced, and new tissue formed at the bed and around the wound.

He presented to the Astria Sunnyside hospital (Sunnyside, WA, USA) with a sacral pressure ulcer measuring 15 cm (length) × 11 cm (width) × 5 cm (depth) present for over 16 years with L4 and L5 exposed spinal segments. Part of the pelvic gridle was removed in the prior hemipelvectomy surgery. L4 and L5 segments were exposed in the wound and L5 segment was significantly debrided to remove nectrotic and/or infected tissue (Figures 1 and 2A). Previously, the wound had been treated with wet-to-dry dressings for 11 years, had shown no signs of healing after three weeks of treatment with negative-pressure wound therapy (NPWT) alone, and had failed multiple skin flap procedures (two rotational and one bilateral). His wound tested positive for methicillin-resistant *Staphylococcus aureus*, *Pseudomonas*, *Escherichia coli*, and *Proteus*. The wound also presented with heavy exudate. The clinical objectives were to manage the infection, reduce the wound exudate, achieve granulation tissue and epithelialization of the wound, and discharge the patient from the hospital. The patient provided written informed consent to be included in the study and to publish photographs. All procedures in this study were in compliance with institutional guidelines and were approved by the review committee.

The synthetic hybrid-scale fiber matrix, Restrata® (Acera Surgical Inc., St. Louis, MO, USA), was placed in complete contact with the wound bed, secured with steri-strips, and applied in conjunction with NPWT to control the wound exudate. The synthetic hybrid-scale fiber matrix was re-applied as needed based on clinician discretion.

Upon the patient’s admittance to the hospital, he was transferred to the acute care center. The wound was not debrided by the clinician because no viable tissue was present to support re-granulation. A 45-day treatment with intravenous (IV) therapy of vancomycin was performed with no healing signs observed. Next, a 12-week IV therapy with meropenem was initiated. In parallel with antibiotic therapy, treatment with the synthetic hybrid-scale fiber matrix was initiated (Figure 2). A total of nine applications of the synthetic hybrid-scale fiber matrix were applied over 11 weeks, as needed, based on clinician discretion. After the first three applications, new granulation tissue covered the exposed bone at the end of the spinal column and filled in the depth of the wound. Moreover, the wound exudate was reduced by approximately 50%, and the wound size was reduced by 20%. Continued follow-up revealed that subsequent applications of the synthetic hybrid-scale fiber matrix resulted in significant healing, wound undermining was resolved, and 2 cm of new epithelium formed at and around the wound site. The application of the synthetic hybrid-scale fiber matrix in conjunction with antibiotics resulted in healing of the rectal fistula. The healing response was satisfactory to the point that the clinician ceased the IV antibiotics treatment at week nine of the 12-week treatment course and switched to oral antibiotics. Overall, the wound demonstrated significant healing, wound exudate was notably reduced, and no complications were observed. The excellent healing response and successful infection management resulted in the patient being discharged from the hospital after 135 days of hospitalization.

## Discussion

In this study, to treat a difficult-to-heal refractory wound, a treatment regimen that utilized a synthetic hybrid-scale fiber matrix was conducted. A pressure ulcer that underwent multiple prior therapies over the past 16 years and failed to heal was treated with nine applications of the synthetic hybrid-scale fiber matrix in conjuction with NPWT over 11 weeks. The treatment was successful in reducing wound size, avoiding complications, and allowing patient discharge.

Current treatment options for pressure ulcers include skin grafts, skin flaps, and wound matrices (allograft, xenograft, and synthetics). However, skin grafts require additional surgery to harvest the skin, which results in donor site morbidity, risk of infection, risk of graft rejection, and higher total cost of care [5,6]. Skin grafting was not appropriate in this setting owing to the infective nature of the wound, unsuitable wound bed, and additional comorbidities of the patient. Multiple skin flap reconstructions were performed in an effort to heal this wound, but all had failed. The pressure ulcer treated in this patient was infected and had heavy exudate, and therefore, biologic wound matrices (allografts or xenografts) were not suitable treatment as they are composed of materials that are subject to enzymatic and bacterial degradation and would likely not persist in the wound site long enough for desired healing outcomes to occur.

Reviewing treatment options, the clinician decided to investigate the use of a novel synthetic hybrid-scale fiber matrix to treat the patient. The synthetic hybrid-scale fiber matrix is resistant to enzymatic degradation, and the synthetic materials used to create the matrix have been shown to have antimicrobial properties [7]. These properties made the matrix an excellent tool to be included in the treatment regimen for the patient. Over the course of the treatment with the synthetic matrix, no new sign of infection was reported, which was an excellent health outcome for a wound that was previously highly infected. Another positive response was the significant reduction of the wound exudate. Moreover, granulation tissue rapidly formed over the exposed bone after only three applications of the matrix. Overall, the synthetic hybrid-scale fiber matrix was able to significantly improve the wound condition and allowed the patient to be discharged from the high-cost setting of the hospital.

## Conclusions

A novel clinical approach utilizing the synthetic hybrid-scale fiber matrix was used to treat a challenging, refractory, 16-year-old pressure ulcer. The treatment led to new epithelial tissue formation, tissue coverage over the exposed bone, infection management, wound exudate reduction, and enabled patient discharge from the hospital. Overall, the excellent healing response achieved suggests that the synthetic hybrid-scale fiber matrix should be included as part of a treatment regimen for difficult-to-heal pressure ulcers, and is worthy of further investigation.
